# Temporal stability of naturally acquired immunity to Merozoite Surface Protein-1 in Kenyan Adults

**DOI:** 10.1186/1475-2875-8-162

**Published:** 2009-07-16

**Authors:** Arlene E Dent, Kiprotich Chelimo, Peter O Sumba, Michele D Spring, Brendan S Crabb, Ann M Moormann, Daniel J Tisch, James W Kazura

**Affiliations:** 1 Case Western Reserve University, Cleveland, OH, USA; 2 Kenya Medical Research Institute, Kisumu, Kenya; 3 Walter Reed Army Institute of Research, Silver Spring, MD, USA; 4 Burnet Institute of Medical Research, Melbourne, Australia

## Abstract

**Background:**

Naturally acquired immunity to blood-stage *Plasmodium falciparum *infection develops with age and after repeated infections. In order to identify immune surrogates that can inform vaccine trials conducted in malaria endemic populations and to better understand the basis of naturally acquired immunity it is important to appreciate the temporal stability of cellular and humoral immune responses to malaria antigens.

**Methods:**

Blood samples from 16 adults living in a malaria holoendemic region of western Kenya were obtained at six time points over the course of 9 months. T cell immunity to the 42 kDa C-terminal fragment of Merozoite Surface Protein-1 (MSP-1_42_) was determined by IFN-γ ELISPOT. Antibodies to the 42 kDa and 19 kDa C-terminal fragments of MSP-1 were determined by serology and by functional assays that measure MSP-1_19 _invasion inhibition antibodies (IIA) to the E-TSR (3D7) allele and growth inhibitory activity (GIA). The haplotype of MSP-1_19 _alleles circulating in the population was determined by PCR. The kappa test of agreement was used to determine stability of immunity over the specified time intervals of 3 weeks, 6 weeks, 6 months, and 9 months.

**Results:**

MSP-1 IgG antibodies determined by serology were most consistent over time, followed by MSP-1 specific T cell IFN-γ responses and GIA. MSP-1_19 _IIA showed the least stability over time. However, the level of MSP-1_19 _specific IIA correlated with relatively higher rainfall and higher prevalence of *P. falciparum *infection with the MSP-1_19 _E-TSR haplotype.

**Conclusion:**

Variation in the stability of cellular and humoral immune responses to *P. falciparum *blood stage antigens needs to be considered when interpreting the significance of these measurements as immune endpoints in residents of malaria endemic regions.

## Background

Individuals living in areas where transmission of *Plasmodium falciparum *is intense and stable develop naturally acquired immunity that is characterized by a high degree of protection against high-density parasitaemia and clinical illness. This immunity develops as a consequence of experiencing multiple episodes of blood stage infection throughout infancy and childhood, and may be lost, or markedly diminished, in the absence of periodic boosting by clinically asymptomatic blood stage infections during adulthood [1]. Adaptive cellular and humoral immune responses to blood stage malaria antigens may be influenced by the intensity and temporal pattern of exposure to infective mosquitoes, the duration and intensity of parasitaemia, the severity of illness, and the degree of immune system maturity [1,2]. Appreciating the contribution of these environmental variables and understanding how they influence discrete immune measurements is complicated by observations that malaria infection can suppress T cell responses while boosting B cell and antibody responses, with age as an important confounder [3,4].

Many studies have examined the relationship between antibody responses to *P. falciparum *merozoite antigens and susceptibility to blood stage infection or mild malaria during childhood. Some, but not all, have reported a significant association of IgG antibody levels to the C-terminal region of MSP-1 with parasitological and clinical phenotypes such as parasitaemia and fever with parasitaemia [5-10]. The decay of anti-malarial IgG antibodies has been examined in Kenyan children who present with acute mild malaria. The half life of IgG3 and IgG1 antibodies to C-terminal region of MSP-1, Apical Membrane Antigen 1, and Erythrocyte Binding Antigen 1 is estimated to be 6.1 to 9.8 days [11]. On the other hand, antibodies to MSP-1_42 _appear to persist for at least four months in residents of hypoendemic regions of the Peruvian Amazon [12]. There have been fewer descriptions of how T cell immunity to blood stage antigens varies over time [13-16], and whether T cell and antibody responses are concordant in the same individuals. An appreciation of this aspect of naturally acquired immunity is important to the identification and interpretation of immune assays that may be used as primary and secondary endpoints in clinical vaccine trials that assess immunogenicity and protective efficacy in malaria endemic populations.

The goal of this study was to advance understanding of the temporal stability of quantifiable immune responses to the C-terminal region of *P. falciparum *MSP-1 by adults who are clinically immune to malaria. Assays that measure immune responses to the 3D7 and FVO alleles of the C-terminal fragment of MSP-1, both of which have been included in recent clinical vaccine trials [17-19], were employed. In addition, in order to determine whether time-related changes in anti-MSP-1 antibody levels reflect changes in functional antibody responses to the broad repertoire of merzoite antigens that affect parasite growth *in vitro*, GIA responses to *P. falciparum *were quantified.

## Methods

### Study population

Ethical approval was obtained from the Institutional Review Board for Human Investigation at University Hospitals Case Medical Center and the Ethical Review Committee at the Kenya Medical Research Institute. Twenty-four healthy asymptomatic adult female (n = 7) and male residents (n = 17) with respective median ages 34 and 44 years (age range 18–79 years) from Kanyawegi sub-location, Nyanza Province, Kenya participated in this study. A total of six blood samples from each individual were collected over a period of nine months. Three venous blood samples (20 ml) were collected at three-week intervals from August to October 2004 during a period of relatively low malaria transmission, and again (three collections at three-week intervals) from April to May 2005 during a period of high malaria transmission. Data collected at each time point included the reported history of recent febrile illness, demographic information (age, gender and family household members) and the level of asexual parasitaemia by blood smear. Data from the 16 participants who provided blood samples at all 6 time points are included in the analysis. All of these individuals remained asymptomatic and reported taking no malaria medication during the nine-month period observation.

### Blood smear examination

Thick and thin blood smears were prepared, fixed in 100% methanol, stained with 5% Giemsa solution, and examined by light microscopy for *P. falciparum*-infected erythrocytes. The density of parasitaemia was expressed as the number of asexual *P. falciparum *per μl blood assuming a leukocyte count of 8,000 per μl.

### IFN-γ ELISPOT

IFN-γ ELISPOT was performed as described previously [13,16,20]. Briefly, peripheral blood mononuclear cells (PBMCs) were separated from heparin anti-coagulated blood by Ficoll-Hypaque gradient centrifugation and incubated for 3.5 days with culture medium alone, 5 μg/ml 3D7 MSP-1_42 _or FVO MSP-1_42 _(provided by Carole Long, Malaria Vaccine Development Unit, NIAID, NIH, Rockville, MD) or 1 μg/ml PHA in microtiter plates coated with 5 μg/ml of primary anti-IFN-γ antibody (Endogen M-700A, Worcester, MA). IFN-γ secreting cells were detected after removal of PBMCs and addition of 0.75 μg/ml of biotinylated secondary anti-IFN-γ antibody (Endogen M-700B) followed by horseradish-peroxidase conjugated strepavidin peroxidase (DAKO PO397) and 1% 3-amino-9-ethyl-carbazole substrate in -0.1 mol/L acetate buffer. Spot Forming units (SFU) were counted by Immunospot Satellite analyzer (Cellular Technology, Cleveland OH). An IFN-γ ELISPOT assay was considered positive if the frequency of SFU for PBMCs incubated with MSP-1_42 _or PHA was significantly greater (p < 0.05) than culture medium alone using a chi-square Fisher's exact test. Only PBMC samples with strong positive IFN-γ responses to PHA were included in the analysis (n = 88).

### IgG antibody measurements

IgG antibodies to recombinant 3D7 (E-TSR) and FVO (Q-KNG) alleles of *P. falciparum *MSP-1_42 _were quantified serologically by ELISA as described previously [21]. Briefly, Immunolon 4 plates were coated with 0.1 μg per ml of the 3D7 or FVO allele of MSP-1_42_. Plasma from nine North American adults never exposed to malaria was used as a negative control. Plasma from four known malaria immune Kenyan adults was pooled to create a positive standard. A standard curve was performed for each plate tested. The value obtained with a 1:50 dilution of the positive pool was designated as 100 arbitrary units (AU), 1:100 dilution as 50 AU, 1:200 dilution as 25 AU, 1:500 dilution as 10 AU, 1:1,000 dilution 5 AU, and 1:2,000 dilution as 1 AU. A four-parameter standard fit curve was constructed from the positive control plasma pool and applied to sample values. Positive values were greater than the mean + 3 SD of the value of the individual negative control plasma samples.

Methods to quantify MSP-1_19_-specific IIA were as described previously [20,22]. Briefly, D10-PfM3' which encodes the MSP-1_19_MAD20/3D7/E-TSR allele and an isogenic D10-PcMEGF parasite line in which the antigenically unrelated *P. chabaudi *ortholog replaces *P. falciparum *MSP-1_19 _were tested in parallel. Ring-stage parasites were synchronized twice by sorbitol lysis and allowed to mature to late trophozoite/schizont stages. Purified parasites were adjusted to 4% haematocrit with 0.5% infected red cells, and 50 μl aliquots were placed in 96-well flat-bottom microtiter plates with an equal volume of 1:5 pre-diluted plasma in culture medium (final plasma dilution 1:10, final volume 100 μl). The same batch of pre-diluted plasma was added to the two parasite lines in the same assay. The cultures were incubated for 26 hours to allow for schizont rupture and merozoite invasion. 25 μl of re-suspended cultures were removed, fixed with 0.25% gluteraldehyde in PBS for 45 min, and placed in 1 μg Hoechst 33342 (HO) stain (Molecular Probes, Eugene, Oregon) in 400 μl 1× PBS for >24 hours at 4°C [23,24]. Stained cells were examined using the UV laser on a BD LSR II flow cytometer to collect data from a minimum of 5 × 10^4 ^cells using Becton-Dickinson FACS Diva 5.01. Ring parasitaemia was calculated by quantifying singly infected erythrocytes plus multiply infected erythrocytes (quantified as having two rings) according to flow cytometry gating previously described [24]. FlowJo 8.5.1 was used to analyse cytometry data. The mean number of ring-stage parasitaemia for duplicate wells was calculated, and results expressed as a percentage of the ring-stage parasitaemia of non-immune control plasma (derived from non-malaria exposed adults) in parallel cultures. The percent change of invasion inhibition attributable to anti-MSP-1_19 _antibodies was calculated by subtracting the percent invasion of D10-PfM3' relative to non-immune controls from the percent invasion of D10-PcMEGF relative to non-immune controls. A positive response was defined as ≥ 5% inhibition attributable to MSP-1_19 _IIA. Overall antibody-mediated GIA was determined from results of the MSP-1_19 _IIA assay using growth of the D10-PfM3' line only. Growth inhibition based on duplicate wells was calculated with the following equation: 100 – (test plasma parasitaemia/non-immune plasma parasitaemia) × 100. Plasma from four malaria naïve US adults was pooled as the non-immune plasma controls. A positive response was defined as ≥ 15% D10 inhibition.

### MSP-119 haplotype determination

DNA was extracted from 200 μl venous blood using QIAamp DNA blood mini kit (Qiagen Corp, Valencia, CA). Methods to quantify the four major MSP-1_19 _haplotypes by PCR/ligase detection reaction fluorescent microsphere assay (PCR/LDR-FMA) were as described previously [25].

### Rainfall data

Monthly rainfall data for the Kisumu area was purchased from the Kenyan Ministry of Transport, Kenya Meteorological Department. Rainfall from July 2004 to July 2005 was recorded as mm per month.

### Statistical analysis

Kappa analyses were conducted using SAS Version 8.2 (Carey, NC).

Quantitative differences between experimental groups were evaluated by paired *t *test. Pearson's correlation was used to compare MSP-1_19_-specific IIA results with serologically determined anti-MSP-1_42 _IgG antibody and responses measured at each time point within each assay. Generalized Estimating Equation (GEE) was utilized to examine repeated immune correlates of protection dichotomous values over time from the same individual. Mixed linear model was utilized to examine repeated immune correlates of protection continuous values over time from the same individual.

## Results

### Dichotomous immune outcomes and temporal stability

Ninety-six determinations (16 individuals examined at six time points) were made for each of the four tests. The percent positive outcomes for each test were 70% for MSP-1_42 _IgG ELISA (3D7 and FVO alleles considered together), 46% for overall GIA, 41% for MSP-1_19_-specific IIA, 67% for T cell 3D7 MSP-1_42 _specific IFN-γ production, and 10% for T cell FVO MSP-1_42 _specific IFN-γ production. T cell responses to FVO MSP-1_42 _were disproportionately low in this population and not evaluated further. Thirty-three percent of the positive immune outcomes were observed coincidental with a *P. falciparum*-positive blood smear.

**Figure 1:**
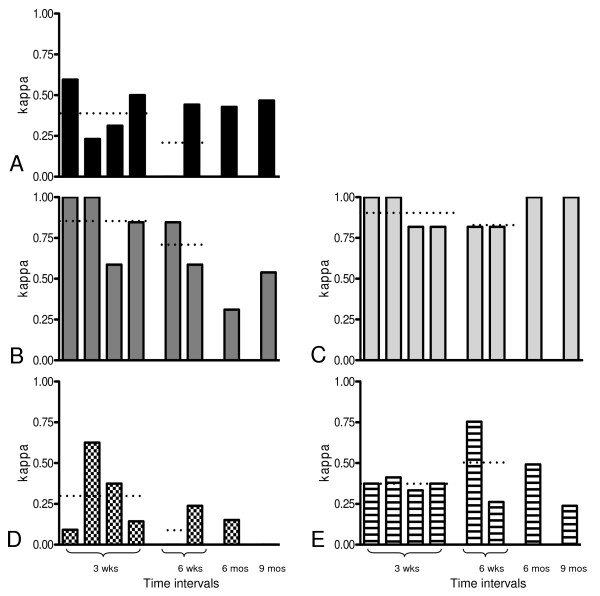
**Temporal stability of five immunologic assays at various time intervals**. X axis: The first group of columns represents assays performed at four different 3-week intervals (Aug. 24 to Sept. 15, Sept. 15 to Oct 6, Apr. 6 to May 5, May 5 to May24). The second group of columns represents assays performed at two different 6-week intervals (Aug. 24 to Oct. 6, Apr. 6 to May 24). The single columns at far right represent assays performed at a single 6-month (Oct. 6 to Apr. 6) and 9-month interval (Aug. 24 to May 24). Horizontal dotted lines across 3-week or 6-week columns indicate average kappa scores. Sample number comparison represented by each column is 16 unless otherwise specified. Y axis: Kappa score: Scores of 0.81–1.0, 0.61–0.80, 0.41–0.60, 0.21–40, and <0.20 indicate, respectively, almost perfect agreement and high stability, substantial stability, moderate stability, fair stability, and slight to no stability. A: 3D7 MSP-1_42 _stimulated INF-γ production measured by ELISPOT. Kappa scores indicate moderate temporal stability over 3-week intervals (0.41) with fair stability (0.22) at 6-week intervals. Sample number for each column from left to right is 15, 10, 11, 12, 14, 16, and 16. B: 3D7 MSP-1_42 _IgG measured by ELISA. Kappa scores indicate high (0.86) and substantial temporal stability (0.72) over 3-week and 6-week intervals. At 6-month and 9-month intervals, stability was fair and moderate. C: FVO MSP-1_42 _IgG measured by ELISA. Kappa scores indicate high to substantial temporal stability for all time intervals tested. The average kappa score for 3-week and 6-week intervals is 0.91 and 0.82. D: MSP-1_19 _IIA. Kappa score at 3-week interval (0.31) indicates fair temporal stability that disappears at longer time intervals. E. Growth Inhibitory Assay (GIA). Kappa scores at 3-week (0.37), 6-week (0.51), and 6-month interval indicate fair to moderate stability. The kappa score at the 9-month interval (0.20) indicated the lowest degree of temporal stability.

The kappa test of agreement between paired samples was used to determine the stability of immune measurements over four time intervals: three weeks, six weeks, six months, and nine months. Immune assay results were expressed as positive or negative using criteria described in the methods section. Kappa test establishes the percent agreement of responses beyond chance such that consistent positive responses and/or consistent negative responses generate higher kappa scores. A kappa score of 0.81–1.00 was regarded as "almost perfect" and considered to reflect consistent positive or negative results between paired samples over a specified time interval. A score of 0.61–0.80 was regarded as "substantial agreement." A score of 0.41–0.60 was regarded as "moderate agreement," a score of 0.21–0.4 as "fair agreement", and a score <0.20 as "slight agreement [26,27]." Figure 1 illustrates the kappa values for each time interval assessed. Each time interval was assessed independently. Kappa scores for 3D7 MSP-1_42 _stimulated IFN-γ production indicate fair to moderate temporal stability over the time intervals examined. In contrast, kappa scores for serologically determined IgG antibody to FVO MSP-1_42 _indicate essentially substantial stability for all time intervals tested. IgG antibody to 3D7 MSP-1_42 _shows good stability over three- and six-week intervals, which decreased over the six-month interval. GIA appears to be moderately stable over time intervals as long as six months. 3D7 MSP-1_19_-specific IIA demonstrates fair stability at three-week intervals that disappears at longer time intervals. In summary, IgG antibody to 3D7 and FVO MSP-1_42 _measured serologically by ELISA were the most stable over time, followed by 3D7 MSP-1_42 _IFN-γ responses and GIA. MSP-1_19 _IIA was the least stable over time with essentially no consistent response lasting more than three weeks.

**Figure 2:**
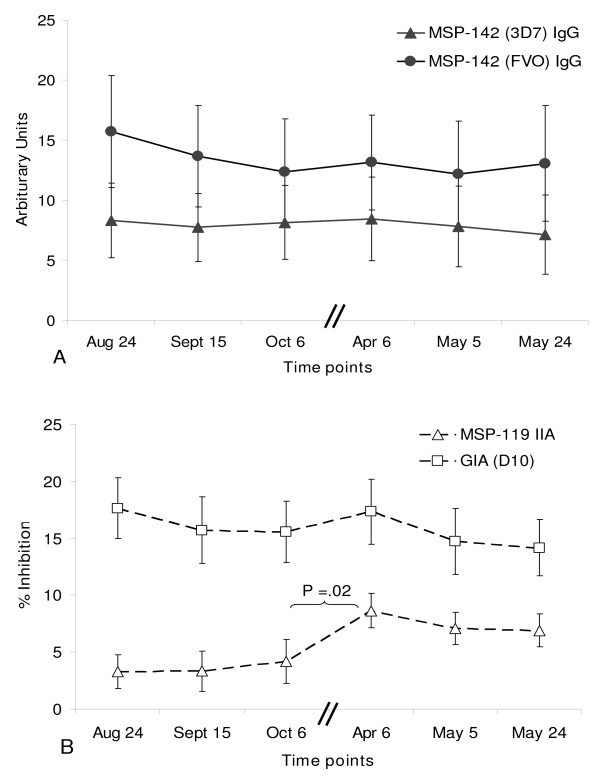
**Temporal stability of quantitative measurements of antibodies**. A: MSP-1_42 _IgG levels (Mean ± SEM) measured by ELISA (solid circles, FVO; solid triangles, 3D7) at the 6 time points. Each time point is separated by approximately 3 weeks with the exception of October 6 to April 6 time points which are separated by 6 months (denoted by two short parallel lines through the x-axis). No significant change in IgG levels was observed over time. B: GIA (open squares) and MSP-1_19 _IIA (open triangles) (Mean ± SEM) at the 6 time points. Between October 6 and April 6 time points, a statistically significant increase in MSP-1_19 _IIA (p = .02, paired *t *test) was observed. No significant change in GIA levels was observed over of any of the other time points.

The GEE final model included categorical data for blood smear positivity, sex, IFN-γ response, GIA, MSP-1_19 _IIA and time intervals, allowing for estimation of stability of repeated observations for each individual. Only MSP-1_19 _IIA showed a significant change from August–October 2004 versus April–May 2005, as illustrated in Figure 2B. No significant differences in MSP-1_42 _IgG antibody determined by serology or T cell IFN-γ responses were seen in these individuals over time using the GEE model.

### Quantitative comparisons

Repeated continuous outcomes from each assay were analyzed with repeated measures models using the PROC MIXED procedure. Time was included as a linear variable. 3D7 MSP-1_42 _IFN-γ, 3D7 and FVO MSP-1_42 _IgG, and GIA responses did not change markedly with time, substantiating results found with GEE analysis. MSP-1_19 _IIA was associated with time increasing 0.1548 units per week (p = 0.003). Results were not affected by sex or age. Little fluctuation over time of 3D7 or FVO MSP-1_42 _IgG antibody was observed (paired *t *tests, Figure 2A). Similarly GIA levels showed little temporal change (paired *t *test). However, MSP-1_19_-specific IIA levels increased significantly (p = 0.02, paired *t *test) in the six-month interval between October 2004 and April 2005 (Figure 2B).

No correlation was observed between 3D7 MSP-1_19 _IIA and 3D7 or FVO MSP-1_42 _IgG antibody determined by serology (Pearson's correlation, R^2 ^= 0.052 and 0.0247 for 3D7 and FVO, respectively). Transgenic *P. falciparum *in which FVO MSP-1_19 _has been replaced by the *P. chabaudi *ortholog have not been constructed. Additionally, there was no correlation between GIA and MSP-1_42 _IgG (R^2 ^= 0.0235 and 0.0036 for 3D7 and FVO, respectively). A modest positive correlation was observed between the functional MSP-1_19 _IIA and GIA results (R^2 ^= 0.1512). This is not surprising since MSP-1_19 _IIA is a component of the overall anti-malarial antibody activity measured by GIA.

Additional materials (Additional Files 1, 2, 3, 4 and 5) demonstrate for each immunologic assay correlation matrices between all the time points and spaghetti plots illustrating the quantitative data over time. While the kappa test takes into account agreement between positive responses and negative responses, correlations are driven only by the magnitude of the positive response. Thus, kappa and correlation tests measure different outcomes. 3D7 MSP-1_42 _IFN-γ correlations ranged from R^2 ^0.001 – 0.81 depending on the time interval examined. 3D7 and FVO MSP-1_42 _IgG correlations ranged from R^2 ^0.001–0.81 and 0.77 – 0.95, respectively. MSP-1_19 _IIA and GIA correlations ranged from R^2 ^0.001–0.55 and 0.2 – 0.69, respectively, depending on the time intervals examined.

### Association of rainfall with blood stage infection, MSP-119 haplotypes, and MSP-119 IIA

**Figure 3:**
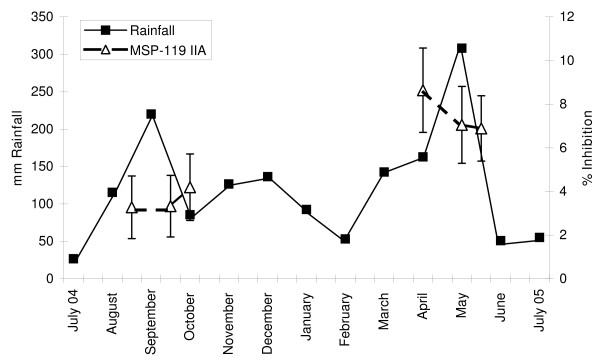
**Kisumu rainfall (mm/month) from July 2004 to July 2005**. The left Y axis indicates mm of monthly rainfall (solid squares) and the right Y axis depicts percent inhibition due to MSP-1_19 _IIA (open triangles). Rainfall is displayed at monthly time points. Peaks in rainfall were observed in September (corresponding to the short rains in western Kenya) and in May (corresponding to the long rains). MSP-1_19 _IIA was measured at approximately 3-week intervals at time points indicated. Higher levels of MSP-1_19 _IIA were observed with increased rainfall.

Rainfall was used to estimate transmission intensity since this climatic variable has previously been found to track with *P. falciparum *transmission in this area of Kenya [28,29]. Cumulative rainfall from August from September 2004 was 336 mm, which was lower than from April to May 2005 when it was 471 mm (Figure 3). Twelve of 48 blood samples obtained during the former period were positive for *P. falciparum *by microscopy compared with 27 of 48 samples during the latter period (p = 0.0018, chi-square test). The average parasite density during both time intervals was <100 asexual parasites/μl blood. None of the participants experienced fever or other symptoms of malaria at any time during the 9-month observation period.

The 19 kDa C-terminal fragment of *P. falciparum *MSP-1 has four predominant alleles based on point mutations resulting in four non-synonymous amino acid changes: E-TSR (3D7/PNG-MAD20 type), E-KNG (Uganda-PA type), Q-KNG (FVO/Wellcome type), and Q-TSR (Indo type). From August to October 2004, 41 of 48 samples were tested for MSP-1_19 _haplotypes. By the MSP-1_19 _PCR/LDR-FMA method, six positive samples had five E-KNG haplotypes and two Q-KNG haplotypes (one sample had both the E-KNG and Q-KNG haplotypes). From April to May 2005, 48 samples were tested. Nine positive samples were detected by MSP-1_19 _PCR/LDR-FMA including three E-TSR, three E-KNG, and three Q-KNG haplotypes. All of those with the E-TSR (3D7) haplotype were found between April and May 2005 which coincided with increased MSP-1_19 _IIA levels (E-TSR/3D7). In contrast, IgG antibody to the E-TSR/3D7 haplotype measured by serology did not increase.

### Association between T cell IFN-γ and humoral immune responses

There was no correlation between T cell IFN-γ responses and antibody responses as determined by correlation coefficients, kappa test, and GEE at individual time points or over time.

## Discussion

MSP-1 is among the most abundant merozoite surface proteins of *Plasmodium *species. It is anchored to the outer membrane of the merozoite by glycophosphatidylinositol, and is considered to be essential to the cyclical process of erythrocyte invasion by malaria parasites. The *msp-1 *gene is conserved in the genomes of all *Plasmodium *species described to date, and the inability to delete the gene is consistent with the notion that the protein has an essential and non-redundant role in blood stage infection [30]. Studies of malaria naïve primates vaccinated with the penultimate and ultimate C-terminal 42 kDa and 19 kDa processed fragments of *P. falciparum *MSP-1 have shown that induction of immunity to this single merozoite ligand can mediate protection against blood stage infection manifest as reduced or absent parasitaemia and reduced severity of anaemia [5,6,8,10]. Experimental subunit vaccines containing recombinant C-terminal fragments of MSP-1 have advanced beyond examination of safety and immunogenicity in malaria naïve humans to studies of immunogenicity and efficacy in malaria endemic populations [19,31-33]. The goal of this study was to characterize the temporal stability of selected immune markers to MSP-1 in Kenya adults with naturally acquired immunity to malaria in order to increase knowledge of whether the temporally unstable and transient nature of antibody responses to MSP-1 (and more generally other blood stage antigens) observed in children extends to adults.

Four immune assays were chosen for study based on previous observations suggesting that they may correlate with protection against parasitaemia and/or clinical morbidity: malaria antigen specific T cell IFN-γ measured by ELISPOT [13,34-37], IgG antibodies to recombinant MSP-1_42 _proteins measured by ELISA [5,6,8,10], MSP-1_19_-specific IIA [20], and GIA [23,38]. Based on serial observations of 16 adult residents of western Kenya examined over a nine-month period, these results show that 1) IFN-γ responses to 3D7 MSP-1_42 _are fair to moderately stable; 2) IgG antibodies to the FVO and 3D7 alleles of MSP-1_42 _measured serologically by ELISA are most stable over time; and 3) functional measures of MSP-1_19 _specific IIA is a transient but potentially sensitive indicator of antibody-mediated immunity that may reflect recent exposure to *P. falciparum *with the corresponding MSP-1_19 _haplotype.

Previous observations indicate that residents of high transmission regions have attenuated cellular responses to *P. falciparum *blood stage antigens in comparison to those living in low transmission regions or who experience few malaria episodes [39,40]. The finding that IFN-γ responses to 3D7 MSP-1_42 _were fair to moderately stable over the time intervals assessed is consistent with this observation. Similarly, in a repeat cross-sectional study of adults and children in western Kenya, IFN-γ T cell responses to LSA and TRAP peptides in adults were not stable over a nine-month time interval [16]. The lack of correlation between humoral and cellular responses to MSP-1 seen here is consistent with previous studies [10]. The parasitological variables and immune mechanisms accounting for the instability of MSP-1 specific T cell cytokine responses are not known. It is possible that malaria infection *per se *may alter T cell responses since blood stage infection has been associated with reduced IFN-γ responses [41]. In the study described here 41% (32/96) of PBMC donor samples were positive for asexual *P. falciparum *by blood smear, although infection status had no correlation with IFN-γ ELISPOT results when analysed by GEE. In addition to extending the current study design to children and infants, a larger sample size may be needed to detect small but significant associations. Future studies that include flow cytometry to measure production of other cytokines in addition to IFN-γ, multifunctional T cells, and T cell memory subsets will be needed to define more clearly the contribution of merozoite antigen-specific T cells in mediating naturally acquired and vaccine-elicited immunity.

The kappa test is an objective evaluation of agreement of an experimentally determined immune variable over defined time intervals. An inherent bias in interpreting the data described here (or any analysis of this type) is the criteria used to determine a positive or negative immune response. Widely accepted criteria to differentiate positive and negative responses for ELISPOT and ELISA assays were used in this study. Functional antibody assays (GIA and MSP-1_19_-specific IIA) can have arbitrary cut-off values to define a positive or negative response. Vaccine studies have often used a GIA cut-off of 15% for positive responders, as the case in this study. On the other hand, cut-off thresholds for MSP-1_19_-specific IIA have not been well defined. In a previous analysis, high levels (upper quartile) of MSP-1_19 _IIA were associated with protection in Kenyan adults living in a highland area where malaria transmission is low and unstable [20]. In contrast, MSP-1_19 _IIA levels are frequently low or absent in Kenyans living in the nearby holoendemic area where the current study was performed (manuscript in preparation).

The lack of correlation between IgG antibodies measured by ELISA and functional antibody assays is not surprising. It is becoming more apparent that functional and serologic assays of antibody responses to merozoite proteins measure different aspects of antibody-mediated immunity in naturally exposed populations [20,38,42]. A strong correlation between ELISA and functional antibody assays is observed in vaccination studies that involve malaria naïve humans [18,43]. In contrast, vaccine studies conducted in malaria endemic regions have not found consistent correlations between ELISA and functional assay responses [44]. Of note with respect to the current observations, GIA and MSP-1_19 _IIA assays had fewer positive responses than did serologically measured responses. This may have resulted in greater temporal stability of results by ELISA but would not have affected quantitative comparisons. Stable ELISA measured antibody responses to MSP-1 have been previously observed in naturally immune adults [32].

The results of this study showing an increased level of MSP-1_19_-specific IIA during periods of increased rainfall and blood stage parasitaemia suggest that this assay may be a transient but nevertheless potentially sensitive indicator of immunity that reflects recent experience with blood stage parasitaemia. Moreover, the finding of an increase in MSP-1_19_-specific IIA suggests that functional as opposed to serologic measurements of antibodies may be important in studying polymorphic vaccine candidate antigens. In this regard, more extensive studies examining functional antibody assays that detect not only erythrocyte invasion by *P. falciparum *bearing MSP-1_19 _FVO (Q-KNG) allele but also other polymorphic merozoite proteins is warranted.

## Conclusion

These data underscore the lack of temporal stability of current assays that measure humoral and cellular immune responses to MSP-1 in adults with naturally acquired immunity to *P. falciparum*. Functional antibody measurements of MSP-1_19_-specific IIA may be a transient indicator of recent haplotype-specific infection. Overcoming the temporal instability of immune responses generated against MSP-1 beyond those demonstrated by clinically immune adults may be necessary to develop a protective blood stage vaccine.

## Abbreviations

IFN: interferon; MSP: merozoite surface protein; IIA: invasion inhibition antibodies; GIA: growth inhibitory activity; PBMC: peripheral blood mononuclear cells; SFU: spot forming units; AU: arbitrary units; HO: Hoechst; PCR/LDR-FMA: PCR ligase detection reaction fluorescent microsphere assay; GEE: generalized estimating equation; ANOVA: analysis of variance.

## Competing interests

The authors declare that they have no competing interests.

## Authors' contributions

AED carried out antibody assays, data analysis and drafted the manuscript. KC carried out cellular assays. POS was responsible for participant recruitment and study coordination. MDS carried out cellular assays and participated in study design. BSC developed assays and manuscript preparation. AMM participated in study conception, study design, and manuscript preparation. DJT performed data analysis. JWK participated in study conception, study design, and helped to draft the manuscript. All authors read and approved the final manuscript.

## Supplementary Material

Additional file 1 **Quantitative data for MSP-1_42 _(3D7) stimulated INF-γ**. Correlation matrices and spaghetti plots for MSP-1_42 _(3D7) stimulated INF-γ at all time points Click here for file

Additional file 2 **Quantitative data for MSP-1_42 _(3D7) ELISA measured responses**. Correlation matrices and spaghetti plots for MSP-1_42 _(3D7) ELISA measured responses at all time points Click here for file

Additional file 3 **Quantitative data for MSP-1_42 _(FVO) ELISA measured responses**. Correlation matrices and spaghetti plots for MSP-1_42 _(FVO) ELISA measured responses at all time points Click here for file

Additional file 4 **Quantitative data for MSP-1_19 _IIA measured responses**. Correlation matrices and spaghetti plots for MSP-1_19 _IIA measured responses at all time points Click here for file

Additional file 5 **Quantitative data for GIA measured responses**. Correlation matrices and spaghetti plots for GIA measured responses at all time points Click here for file
