# Supplementary material for: Temporal stability of naturally acquired immunity to Merozoite Surface Protein-1 in Kenyan Adults
Source: Malar J. 2009 Jul 16;8:162. doi: 10.1186/1475-2875-8-162 (PMC2719655; doi:10.1186/1475-2875-8-162)

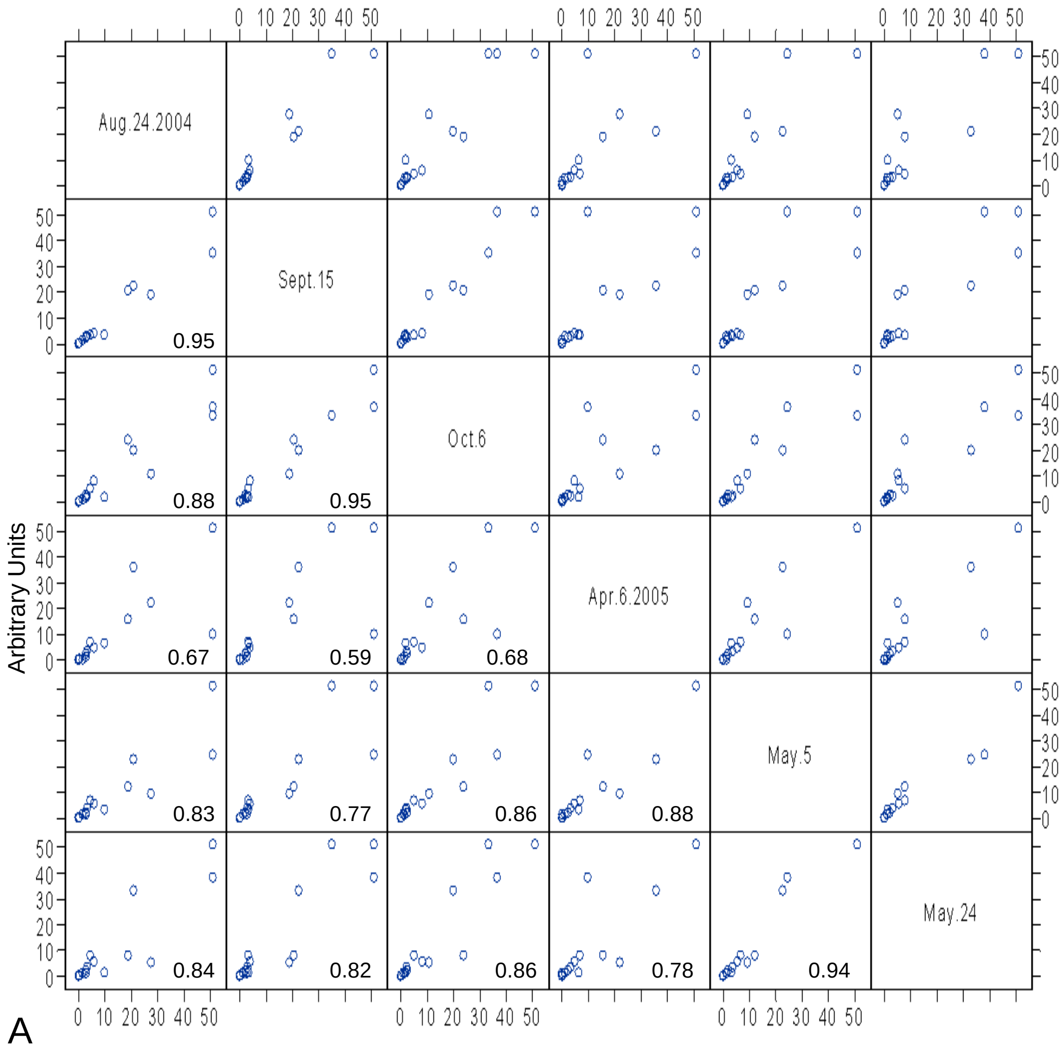


Supplementary Figure 3: A: MSP-$1_{42}$ (FVO) ELISA measured responses compared between all time points with Pearson's correlation $R^2$ value in the bottom right corner of each comparison. B: MSP-$1_{42}$ (FVO) ELISA data for each individual at each time point.

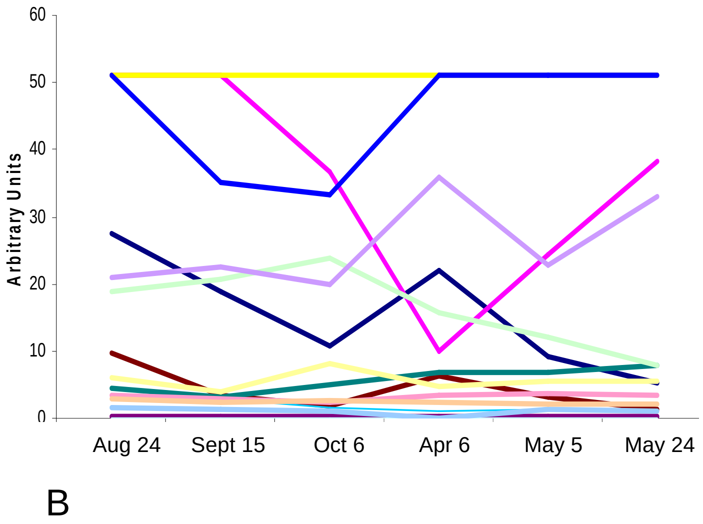

Supplement: Additional file 3 — Quantitative data for MSP-142 (FVO) ELISA measured responses. Correlation matrices and spaghetti plots for MSP-142 (FVO) ELISA measured responses at all time points [file 1475-2875-8-162-S3.pdf]
