# Supplementary material for: Temporal stability of naturally acquired immunity to Merozoite Surface Protein-1 in Kenyan Adults
Source: Malar J. 2009 Jul 16;8:162. doi: 10.1186/1475-2875-8-162 (PMC2719655; doi:10.1186/1475-2875-8-162)
Supplement: Additional file 5 — Quantitative data for GIA measured responses. Correlation matrices and spaghetti plots for GIA measured responses at all time points [file 1475-2875-8-162-S5.pdf]

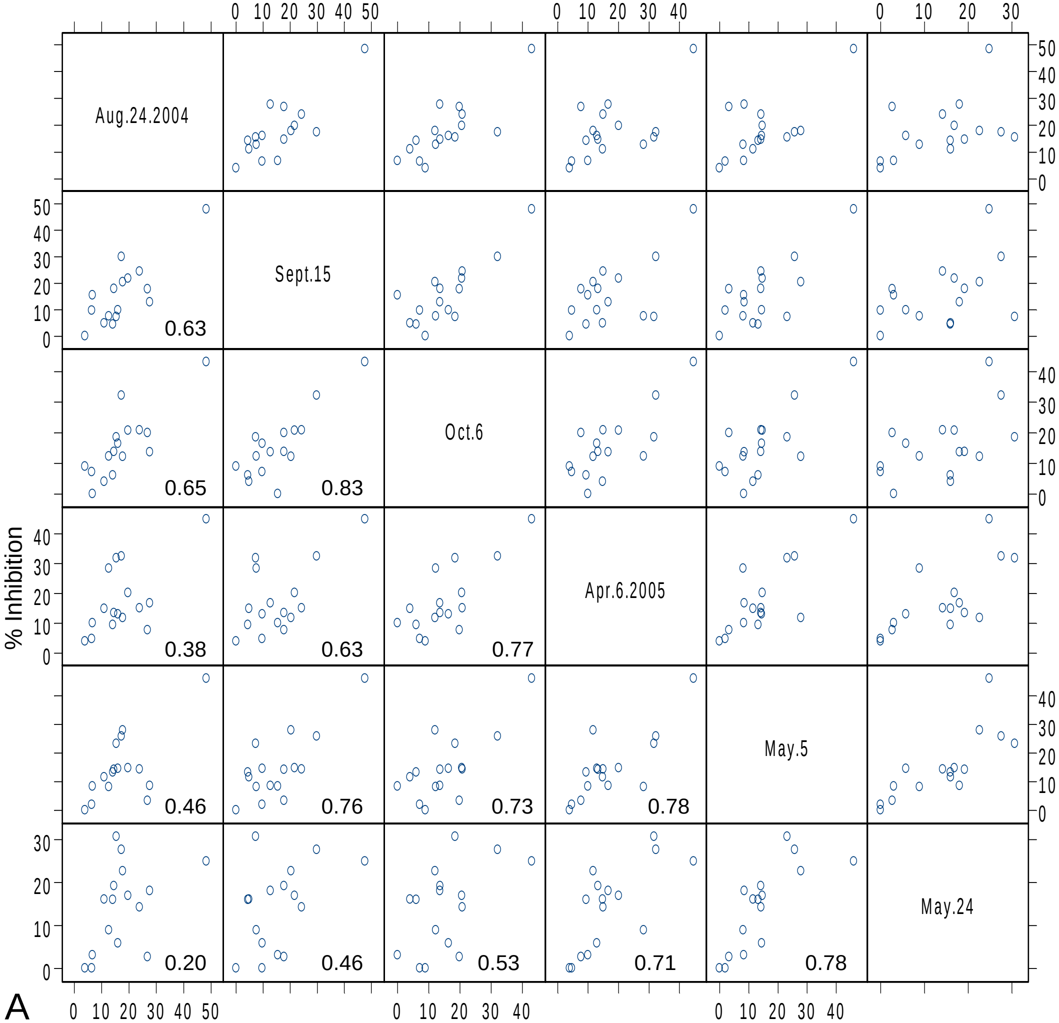

A

Supplementary Figure 5: A: GIA measured responses compared between all time points with Pearson's correlation R^2 value in the bottom right corner of each comparison. B: GIA data for each individual at each time point.

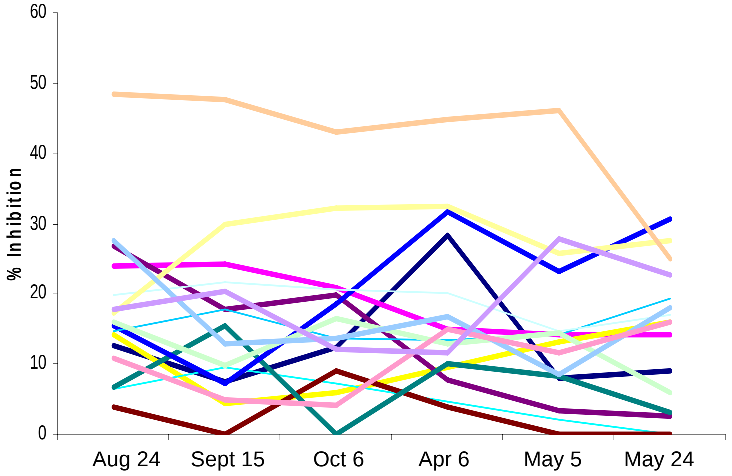

B
